# Supplementary material for: Evaluation of the Biological Effect of Non-UV-Activated Bergapten on Selected Human Tumor Cells and the Insight into the Molecular Mechanism of Its Action
Source: Int J Mol Sci. 2023 Oct 25;24(21):15555. doi: 10.3390/ijms242115555 (PMC10647757; doi:10.3390/ijms242115555)
Supplement: Supplementary file 1 [file ijms-24-15555-s001.zip › supplementary Table S1 - Cell lines data.pdf]

**Table S1. Summary of data on cell lines used in experiments**

| | CELL LINE | HOS | Saos-2 | HT29 | Sw620 | RPMI8226 | U266 |
|---|---|---|---|---|---|---|---|
| Phenotypic characteristic | **Tumor source** | primary | primary | primary | metastasis lymph node | primary | primary |
| | **Histology** | osteosarcoma | osteosarcoma | colon carcinoma | colon adenocarcinoma | plasmocytoma myeloma | plasmocytoma myeloma |
| | **Morphology** | epithelial-like | epithelial-like | epithelial | epithelial | limphoblastic | limphoblastic |
| | **Growth Mode** | adherent | adherent | adherent | adherent | non-adherent | non-adherent |
| | **Migration/ Invasiveness *(in vitro)*** | +++ | ++ | ++ | +++ | + | + |
| Gene mutation | *APC* | - | - | + | + | - | - |
| | *BRAF* | - | - | + | - | - | + |
| | *CDKN2A* | + | - | - | - | - | - |
| | *EGFR* | - | - | - | - | + | - |
| | *PIK3CA* | - | - | + | - | - | - |
| | *KRAS* | - | - | - | + | + | - |
| | *SMAD4* | - | - | + | + | - | - |
| | *TP53* | + | + | + | + | + | + |
| | *RB1* | - | + | - | - | - | + |

*Abbreviations:* APC (Adenomatous polyposis coli), BRAF ( V-Raf Murine Sarcoma Viral Oncogene Homolog B ), TP53 (Tumor protein p53), RB1 (Retinoblastoma1/RB transcriptional corepressor 1), CDKN2A (Cyclin-dependent kinase inhibitor 2A)/tumor suppressor gene.

**References:**


64. Lauvrak, S.U.; Munthe, E.; Kresse, S.H.; Stratford, E.W.; Namløs, H.M.; Meza-Zepeda, L.A.; Myklebost, O. Functional characterization of osteosarcoma cell lines and identification of mRNAs and miRNAs associated with aggressive cancer phenotypes. *Br. J. Cancer*. **2013,** *109(8)*, 2228-2236. doi: 10.1038/bjc.2013.549

65. Zhu, X.L.; Liang, L.; Ding, Y.Q. [Expression of FMNL2 and its relation to the metastatic potential of human colorectal cancer cells]. *Nan Fang Yi Ke Da Xue Xue Bao*. 2008 28(10), 1775-1778. Chinese. PMID: 18971169.

66. Jiang, S.; Zhou, F.; Zhang, Y.; Zhou, W.; Zhu, L.; Zhang, M.; Luo, J.; Ma, R.; Xu, X.; Zhu, J.; Dong, X.; Zhang, S.; Fang, J.; Sun, J.; Yang, X. Identification of tumorigenicity-associated genes in osteosarcoma cell lines based on bioinformatic analysis and experimental validation. *J. Cancer.* **2020**, *11(12)*, 3623-3633. doi: 10.7150/jca.37393

67. https://cancer.sanger.ac.uk/cosmic; Catalogue of Somatic Mutations in Cancer (assec on; 17 October 2023)

68. https://maayanlab.cloud/Harmonizome/dataset/COSMIC+Cell+Line+Gene+Mutation+Profiles COSMIC Cell Line Gene Mutation Profiles (acces on; 17 October 2023.)
