# Supplementary figures and images for: Evaluation of the Biological Effect of Non-UV-Activated Bergapten on Selected Human Tumor Cells and the Insight into the Molecular Mechanism of Its Action
Source: Int J Mol Sci. 2023 Oct 25;24(21):15555. doi: 10.3390/ijms242115555 (PMC10647757; doi:10.3390/ijms242115555)

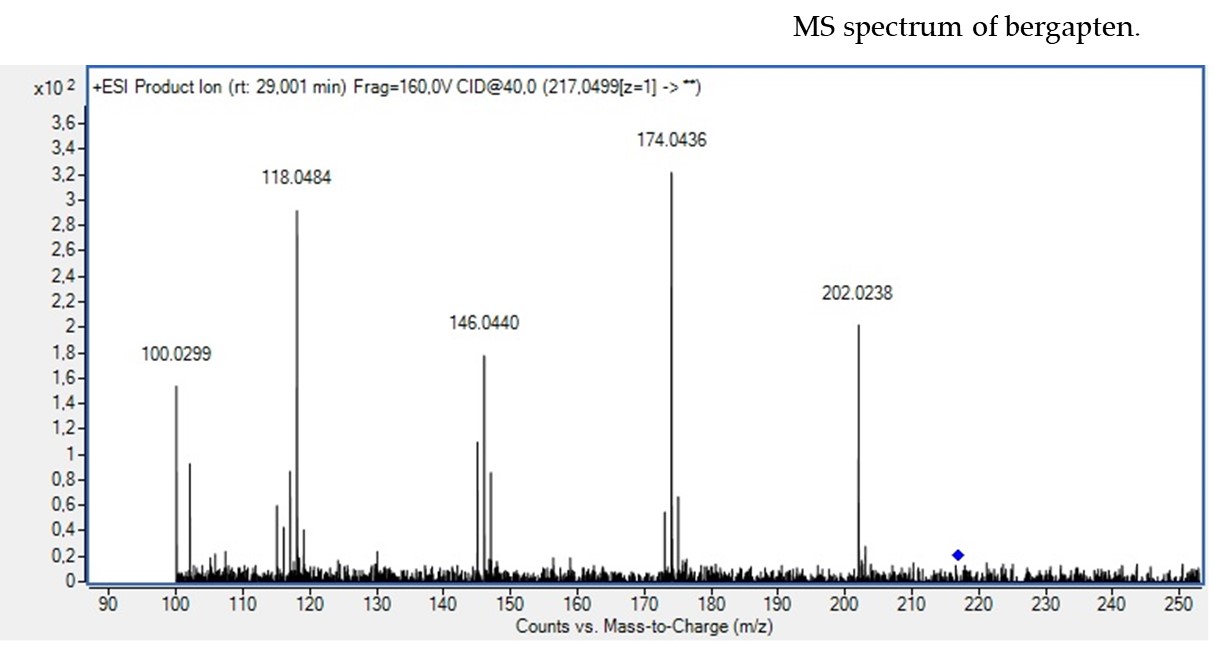

Supplement: Supplementary file 1 [file ijms-24-15555-s001.zip › Supplementary Figure S1_MS spectrum of 5MOP.jpg]

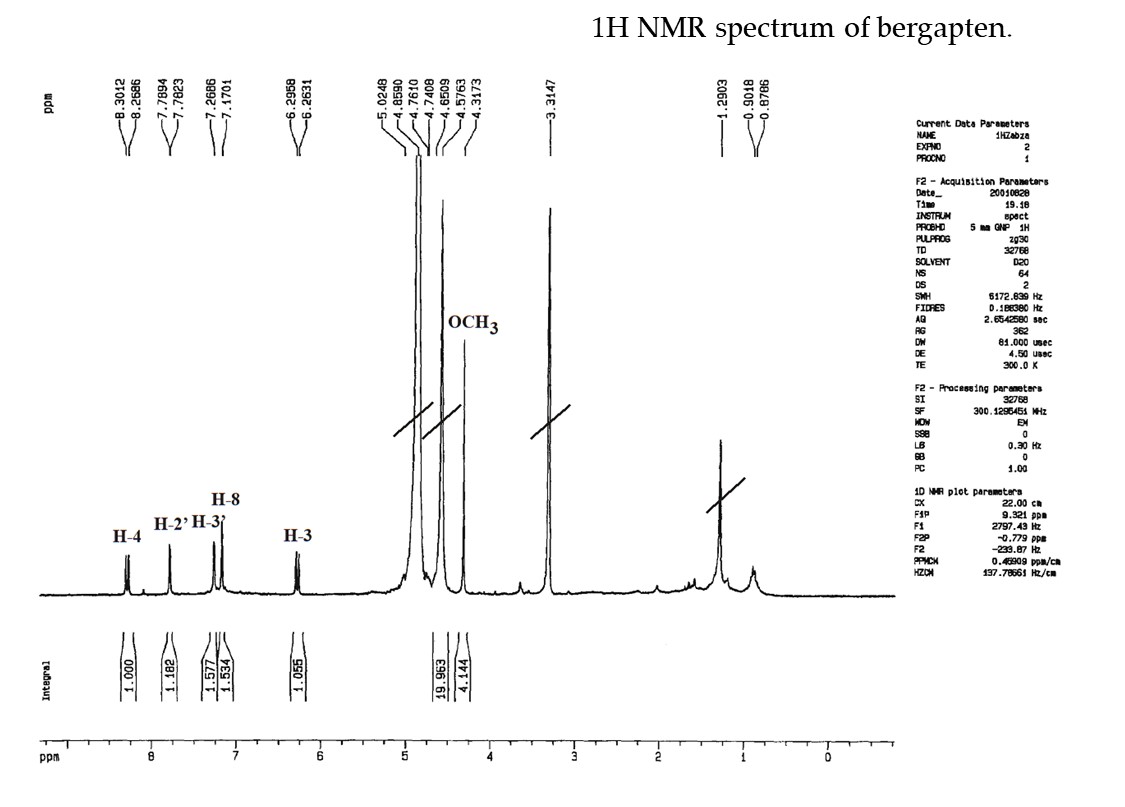

Supplement: Supplementary file 1 [file ijms-24-15555-s001.zip › Supplementary Figure S2_1H NMR spectrum of 5MOP.jpg]

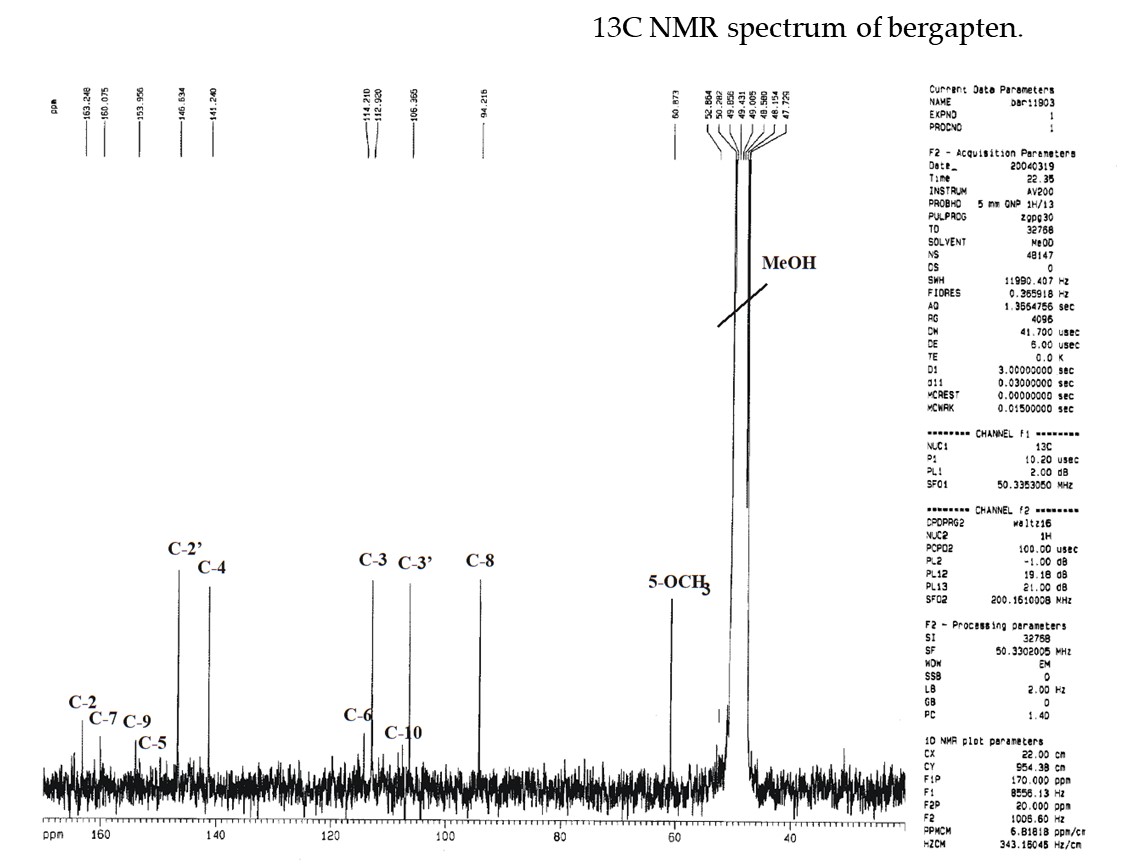

Supplement: Supplementary file 1 [file ijms-24-15555-s001.zip › Supplementary Figure S3_13C NMR spectrum of 5MOP.jpg]

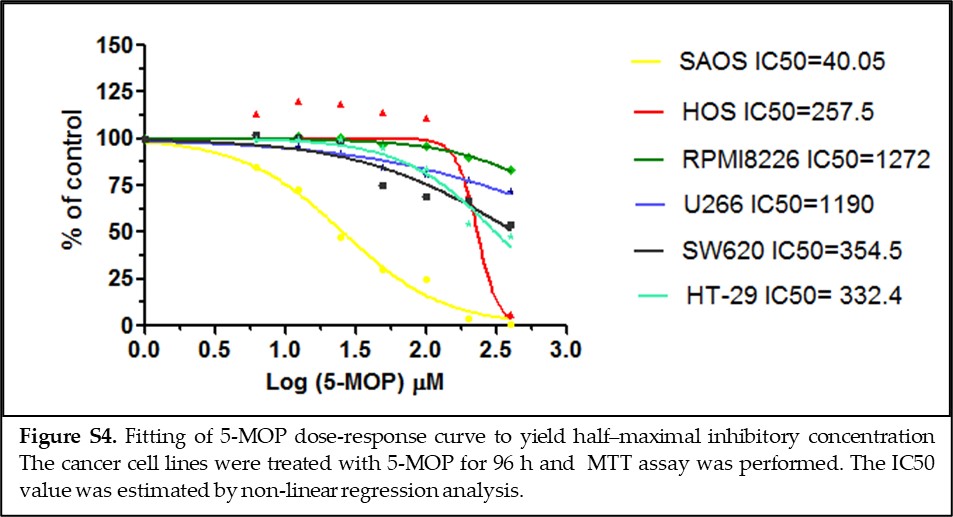

Supplement: Supplementary file 1 [file ijms-24-15555-s001.zip › Supplementary Figure S4.jpg]

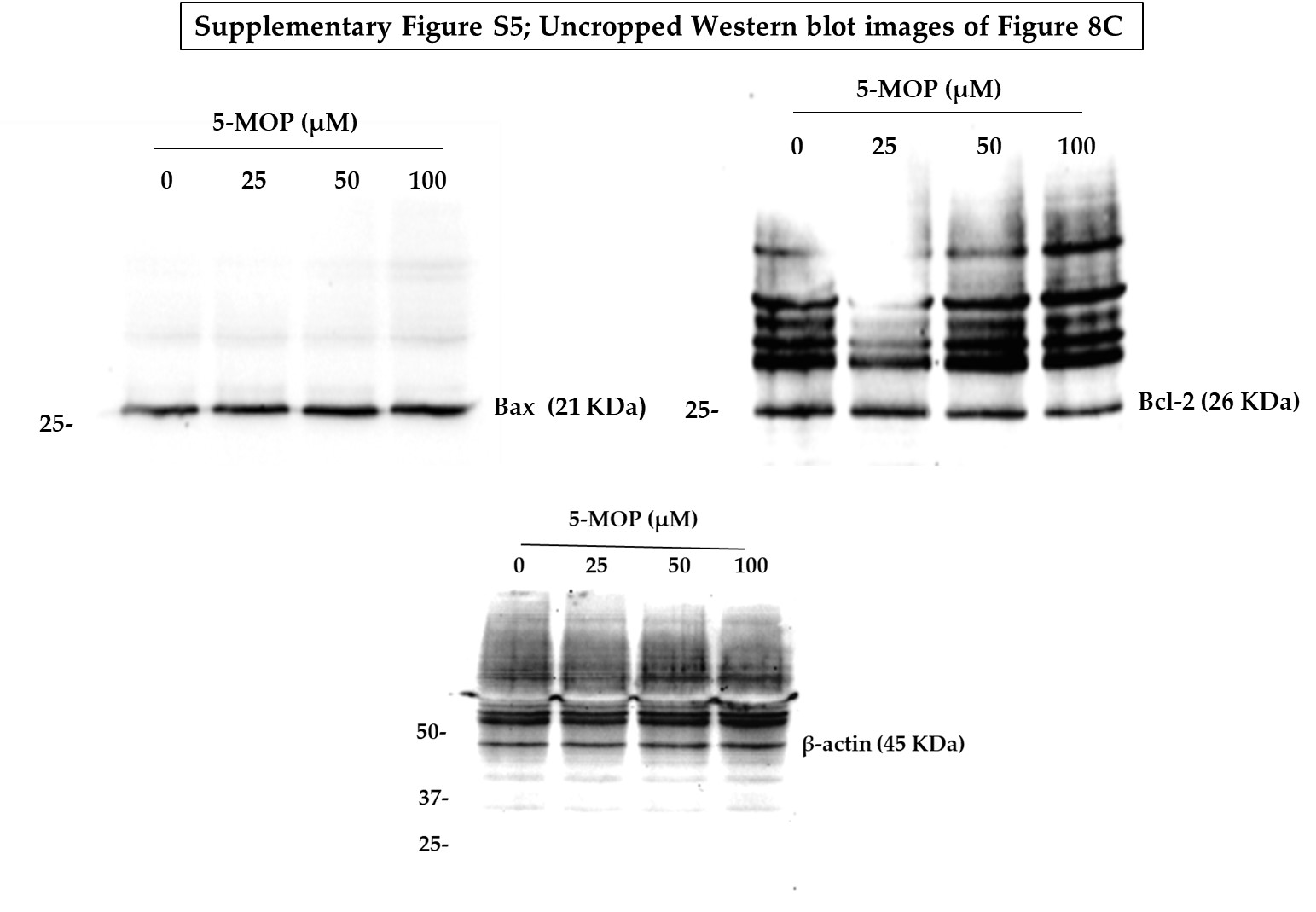

Supplement: Supplementary file 1 [file ijms-24-15555-s001.zip › Supplementary Figure_S5 - Uncropped Western blot images of Fig. 8C.jpg]
